# Physical fitness, hormonal, and immunological responses during prolonged military field training

**DOI:** 10.14814/phy2.13850

**Published:** 2018-09-05

**Authors:** Tommi Ojanen, Petri Jalanko, Heikki Kyröläinen

**Affiliations:** ^1^ Finnish Defence Research Agency Finnish Defence Forces Järvenpää Finland; ^2^ Biology of Physical Activity University of Jyväskylä Jyväskylä Finland; ^3^ National Defence University Helsinki Finland

**Keywords:** Hormonal, immunological, Physical performance, strength, warfighter

## Abstract

Physical fitness is crucial to warfighters’ performance in the battlefield. Previous studies have shown negative changes in their hormonal and neuromuscular responses induced by military field training (MFT). The purpose of this study was to investigate the changes in hormonal and immunological values and body composition during a prolonged MFT and to find out how warfighters’ physical condition influences these changes. Conscripts (*n* = 49, age 20 ± 1 years, height 179 ± 9 cm, body mass 73.8 ± 7.8 kg, fat 12.6 ± 3.7% and BMI 23 kg/m²) were measured before, during, after MFT, and after a 4‐day recovery period. Serum insulin‐like growth factor‐1 (IGF‐1), tumor necrosis factor alpha (TNF‐*α*), interleukin‐6 (IL‐6) concentrations, creatine kinase (CK) activity and leptin concentration were analyzed as well as body composition throughout MFT. Neuromuscular performance was assessed via lower and upper body muscle endurance at the beginning of the study. During MFT, there was a significant decrease (*P* < 0.05) in body mass (2.3%), fat mass (7.7%) and in muscle mass (2.2%), but all of these values recovered to PRE‐levels after the recovery period. Serum IGF‐1 (22%) and leptin decreased (66%) while CK increased (88%) significantly (*P* < 0.05) during MFT but recovered at the end of MFT. Upper body dynamic and trunk isometric muscular endurance had a positive correlation (*r* = 0.37. *P* < 0.05) with the change in IGF‐1 during MFT and a negative correlation with the changes in CK (−0.34, *P* < 0.05). The results show that there were negative changes in conscript's body composition and hormonal and immunological values during the prolonged MFT. These changes suggest that the physiological stress was high during MFT. High levels of upper body and trunk muscular strength were negatively correlated with warfighters’ physiological effects and should therefore be developed prior to actual deployment to reduce the physical decline experienced during prolonged MFTs.

## Introduction

There are multiple challenging stressors that influence warfighters’ physical performance in the battlefield. These stressors include physical strain (Vicente et al. 2013; Pihlainen et al. 2014), exertional fatigue and sleep deprivation, energy deficit (Kyröläinen et al. 2008; Margolis et al. 2014), external load from equipment carried, (Patton et al. 1991; Knapik and Reynolds 2011) and climatic circumstances (Sawka et al. 2007; Caldwell et al. 2011; Nindl et al. 2013). All these stressors are challenges to warfighters in the battlefield and can lead to decreased physical performance or even overreaching.

Previous studies have shown changes in warfighters’ body composition, physical performance, and physiological responses. Body mass has been reported to decline during short military field training (MFT) (≤5 days) by 2.3–5.0% (Rintamäki et al. 2005; Nindl et al. 2002) and by 8% during longer MFT (14 days) (Chester et al. 2013). Nindl et al. (2007) reported a 12.6% reduction in body mass during an 8‐week physically strenuous US Army Ranger ‐course. Sporis et al. (2014) found a 3.6% decline in body mass during a special force training of the Croatian Army. Similar declines can be found also in neuromuscular performance during MFT. Chester et al. (2013) found a decrease of 10% in countermovement jump performance after a 10‐day MFT. Similar findings have also been observed in a 3‐day MFT study by Nindl et al. (2002). Hackney et al. (1991) and Guezennec et al. (1994) have reported an 8% decline in aerobic endurance after a 5‐day strenuous MFT. Even more severe drastic declines in maximal strength performance have been reported after special force training courses (Nindl et al. 2007; Sporis et al. 2014).

IGF‐1 affects the metabolism and growth of cells. Increased concentration of IGF‐1 in blood circulation has been found to be positively influence several health factors, including aerobic endurance, bone thickness, muscle growth and brain function (Friedl 2003). According to Nindl (2009), IGF‐1 concentration in the blood can be used to evaluate warfighters’ physical strain together with other variables. Previous studies have shown a decrease in IGF‐1 values by 24% after MFT with an energy deficit and sleep deprivation (Nindl et al. 2003). Alemany et al. (2008) reported a 50% decline in IGF‐1 values after an 8‐day physically strenuous MFT. During the 8‐week US Army Ranger course, Friedl et al. (2000) and Nindl et al. (2007) observed 50–55% reductions in IGF‐1 values.

Leptin is found to reflect energy deficit (Ahima et al. 1996) and can also be shown to decrease during intense physical strain (Jürimäe et al. 2003) due to increased energy requirements. Gomez‐Merino et al. (2003) found a decrease of 73% in leptin values after a 5‐day MFT with energy and sleep deficit. According to Jürimäe et al. (2011), leptin might be a good tool to evaluate recovery state during training.

Inflammatory indicators have been shown to increase during MFT (Gomez‐Merino et al. 2002, 2003; Chester et al. 2013; McClung et al. 2013; and Margolis et al. 2014). Jürimäe et al. (2011) suggested that an increase in TNF‐*α* concentration could be used as a marker of individuals’ recovery state. IL‐6 has been shown to remain unchanged (Chester et al. 2013) or increase (Gomez‐Merino et al. 2003; McLung et al. 2013) during MFT. No changes in IL‐6 concentrations were observed during Israeli army basic training (Nindl et al. 2012). However, these studies did not report the associations between IL‐6 concentration and physical performance of warfighters. CK activity has been shown to increase during a US Army 14‐day training period by 6‐fold (Kenney et al. 2012). Similar findings have been reported during a 15‐day military survival training (Chester et al. 2013) and winter training (Margolis et al. 2014). Hartmann and Mester (2000) stated that increased CK activity together with decreased physical performance can predict overreaching.

Modern battlefields are challenging for warfighters′ physical performance. If a warfighter has to continue their mission for a prolonged period of time, there is a possibility of overreaching or even overtraining developing. Hormonal and immunological measurements can provide an indication of possible warning signs of overreaching in advance which could be used to control the total physical strain of a warfighter. The current study investigated the changes in hormonal and immunological values and body composition during a prolonged MFT. An additional purpose of the study was to find out how physical condition influences these changes in field conditions. A better knowledge of these associations may help to understand what kind of training warfighters should do for improving their ability to maneuver in the battlefield. It was hypothesized that there would be declines in body mass, skeletal muscle mass, fat mass and also in IGF‐1 and leptin concentrations while there would be increases in CK, IL‐6 and TNF‐*α* concentrations. It was also hypothesized that high levels of endurance would attenuate the decline in IGF‐1 and leptin concentrations and the increases in CK, IL‐6, and TNF‐*α*.

## Methods

### Subjects

Sixty‐One Finnish Army male conscripts volunteered as subjects for the present study. Forty‐nine conscripts completed the study. Twelve individuals dropped out during the study due to discomfort of having to provide blood samples and a lack of motivation to participate in the study. The subjects were 19‐22‐year‐old male conscripts. Mean (±SD) age was 20 (±1) years, height 178.5 (±6.4) cm, body mass 73.5 (±8.7) kg, and body fat 12.6 (±5.0).

Before the study, all the conscripts were fully informed of the experimental design and the possible risks that could be associated with it. Subjects were informed that they could drop out of the study which was a part of their compulsory conscript military training at any stage if they so wished without any consequences. Every subject read and signed an informed consent document before the study commenced. This study was conducted according to the provisions of the Declaration of Helsinki and was granted ethical statement by the Ethics Committee of Central Finland Health Care District. The study was also approved by the Finnish Defence Forces.

### Experimental design

A week before MFT, all the subjects were tested for baseline measurements (PRE). The same tests were also performed at the midway stage of MFT (day 13) (MID), in the end of MFT (day 22) (POST) and after four (4) day (day 26) recovery period (RECO). Body composition, blood samples, and physical fitness tests were measured during these days. Daily diaries were collected to follow the workload and mood states of the conscripts. The entire MFT period was performed in a field condition with three phases. In the first phase, the subjects performed shooting exercises with live ammunition. The objective was to improve their shooting skills and advance their weapon handling abilities, not to exhaust them but rather to ensure that each of them maintained a high level of performance by ensuring that they had appropriate rest. In the second phase, they practiced moving from their base to their attacking positions for the last phase when they executed their mission as a part of a larger scale military exercise. After the prolonged MFT, the subjects had 4 days of recovery time, two at home and two at the garrison before the final measurements were taken. During MFT, the weather was cloudy and windy with occasional rain, temperatures were between 5.1 and 13.7°C, and load carriage varied from 27 to 35 kg depending on individual tasks.

### Measurements

#### Body composition

Body mass (BM), muscle mass (SMM), and fat mass (FM) were determined by using bioelectrical impedance analysis (BIA) (InBody 720, Biospace Co., Ltd., Seoul, South Korea). The measurements were taken after an overnight fast, in the morning between 06:00 and 07:00. The subjects were instructed not to eat anything after their evening meal which was around 19:00. The BIA estimates of body composition have shown to highly correlate with the dual‐energy X‐ray absorptiometry (DXA) method (*r* = 0.82–0.95) (Sillanpää et al. 2014).

#### Maximal cardiovascular performance

Maximal cardiovascular performance was measured by using a 12‐min running test (Cooper 1968). This test has been shown to be valid and reliable for young adults (*r* = 0.87, *μ* = 0.96) (Penry et al. 2011). The running distance was converted to *V*O_2max_ with the following formula: (distance in meters −504.9)/44.73) (Cooper 1968). Before the test, safety instructions were informed to the conscripts and after that they had 15 min time to complete a warm‐up.

#### Standing long jump and muscle endurance tests

Standard Finnish army standing long jump and muscle endurance tests were measured before MFT. The tests consisted of standing long jump, sit‐ups and push‐ups (see more details from Santtila et al. 2006). The participants were instructed to perform as many repetitions in sit‐ups and push‐ups as they were able to perform during 60 sec. There was a recovery period of at least 5 min between the tests. Each participant was instructed to perform the correct performance technique before each test. Only the completed trials with appropriate technique were accepted for final results. Standing long jump have been shown to be highly repeatable (ICC = 0.95) and valid test (*λ *= 0.75) for measuring explosive strength (Markovic et al. 2004). The corresponding reproducibility has also been shown for sit‐ups (ICC = 0.93–0.95) and push‐ups (ICC = 0.83–0.93) (Augustsson et al. 2009).

#### Serum hormone concentrations

Venous blood samples were drawn five times from the antecubital vein after an overnight fast between 06:30 and 07:30 to use immunoassay system for analyzing insulin‐like growth factor‐1 (IGF‐1), interleukin 6 (IL‐6) (Siemens Immulite 2000 XPI, Siemens Healthcare Diagnostics Products Ltd., Gwynedd, UK), tumor necrosis factor alpha (TNF‐*α*) (Siemens Immulite 1000, Siemens Healthcare Diagnostics Products Ltd., Gwynedd, UK), leptin (ELISA‐kit, BioVendor, Brno, Czech Republic/Dynex DS 2, Dynex Technologies, Chantilly), and photometric system for analyzing creatine kinase (CK) levels (Konelab 20 XTi). The sensitivity and interassay variance for these assays were 2.65 nmol/L and 7.6% for IGF‐1, 0.11 pg/mL and 17.6% for IL‐6, 0.19 pg/mL and 9.6% for TNF‐*α* and 3.2 U/L and 5.8% for CK. The samples were centrifuged (Megafire 1.0 R Heraeus, DJB Lab Care, Germany) at 2000 g for 10 min and frozen and transported to laboratories at the University of Jyväskylä for later analysis.

#### Questionnaires

Questionnaires were used to collect information concerning the amount of sleep, stress levels, fatigue, and several other factors. Each morning, the conscripts were given a new diary to complete and the following morning the previous diary was collected and a new one issued. The subjects were asked to write down how many hours they slept each night to the nearest a half an hour. The ratio of perceived exertion (RPE) were measured on a scale of 6–20. Six refers to very light and twenty meaning very heavy physical exertion (Borg 1998).

### Physical activity

Physical activity was monitored using accelerometers (Hookie AM20, Traxmeet Ltd, Espoo Finland). The device was attached to the waist with an elastic belt. The validity (CC = 0.96) of the device has been reported by Aittasalo et al. (2015). The device calculated the total number of steps taken during each day. The subjects were instructed to keep the accelerometer on them at all times.

### Statistical analysis

The data for the present study was analyzed using SPSS Statistics 22 program. For calculating means, standard deviations, and Pearson product moment correlation coefficients conventional statistics were used. The data was analyzed using multivariate analysis of variance (MANOVA) with repeated measures. Probability adjusted *t* tests were used for pairwise comparisons when appropriate. A general linear model, with repeated measures ANOVA was used to analyze the differences between the different measuring points. Bivariate correlation was used for correlation analysis where the changes in the variables between the different time points were tested. The *P *<* *0.05 criterion was used for establishing the statistical significance.

## Results

### Physical performance, activity, and questionnaires

Mean (±SD) estimated *V*O_2max_ was 49.2 ± 4.8 mL/min/kg and standing long jump was 2.29 ± 0.21 m. Subjects performed 40 ± 13 push‐ups and 46 ± 9 sit‐ups in 60 sec. Average sleep time was 6 ± 1 h per day during the prolonged MFT. Mean (±SD) RPE‐score was 9 ± 2, with the highest value at the 13th (12 ± 3) and the lowest value at the 15th (7 ± 1). The subjects took on average 12165 ± 2381 steps per day during MFT.

### Body composition

The declines in BM, SMM, and FM after MFT were all significant, and the values recovered during the 4‐day recovery period, except BM and SMM which did not return to the PRE‐values. BM declined from 70.5 ± 7.1 to 68.9 ± 7.2 kg but recovered to 70.0 ± 7.2 kg as shown in Table 1. The same trend was found in SMM and FM.

**Table 1:** Changes in body composition during MFT

	PRE	MID	POST	RECO
Body mass (kg)	70.5 ± 7.1	70.0 ± 7.5**	68.9 ± 7.2*** ^,^ ††	70.0 ± 7.2* ^,^ ‡‡
Skeletal muscle mass (kg)	35.0 ± 3.5	34.6 ± 3.5	34.2 ± 3.3†	34.5 ± 3.5‡‡
Fat mass (kg)	9.1 ± 5.0	8.9 ± 4.9	8.4 ± 4.7** ^,^ †	9.3 ± 4.7**

*P < 0.05; **^,††, ‡‡^ *P* < 0.01; ***P < 0.001; *^,^**^,^***compared to the PRE values; ^†,^^††^compared to the MID values; ^‡‡^compared to POST values.

### Hormonal and immunological changes

Serum IGF‐1 concentration decreased by 21.5% (*P* < 0.001) between the PRE and MID measurements and almost returned to the PRE‐levels in the RECO measurements (*P* < 0.001). Serum TNF‐*α* concentration decreased by 31.1% (*P* < 0.01) between MID and POST and increased again between POST and RECO almost back to the PRE‐levels (*P* < 0.001). Serum CK increased by 88.0% (*P* < 0.001) between the PRE and MID measurements and decreased (*P* < 0.001) back to PRE‐values in the RECO measurements. Serum leptin concentration decreased by 66.0% (*P* < 0.001) between PRE and MID and increased (*P* < 0.001) almost back to the PRE‐values in the RECO measurement (Table 2).

**Table 2:** Changes in hormonal and immunological concentrations and CK activity during MFT

	PRE	MID	POST	RECO
IGF‐1 (pmol/L)	40.5 ± 7.8	31.8 ± 8.4***	32.2 ± 7.6*** ^,^ †††	38.9 ± 7.7‡‡‡
TNF‐*α* (ng/mL)	9.4 ± 1.9	10.3 ± 3.7**	7.1 ± 1.7***^,^ ††	8.5 ± 1.6* ^,^ †† ^,^ ‡‡‡
Leptin (ng/mL)	3.8 ± 2.8	1.3 ± 1.1***	2.1 ± 1.6*** ^,^ ††† ^,^ ‡‡‡	3.4 ± 3.0††† ^,^ ‡‡‡
IL‐6 (ng/mL)	1.8 ± 2.8	2.0 ± 4.9	1.4 ± 2.1	1.2 ± 2.2
CK (U/L)	106 ± 95	198 ± 88***	141 ± 63* ^,^ †††	107 ± 40††† ^,^ ‡‡‡

*P < 0.05; **^,††,‡‡^ *P* < 0.01; **^,†††,‡‡‡^ *P* < 0.001; *^,^**^,^***compared to the PRE values; ^††,†^^††^compared to the MID values; ^‡‡‡^compared to POST values.

### Associations between physical performance and body composition and changes in immunological and hormonal concentrations

The number of push‐ups was positively correlated with the absolute (*r* = 0.34, *P* < 0.05) and relative (*r* = 0.37, *P* < 0.05) changes in the IGF‐1 concentration and negatively with the absolute (*r* = −0.32, *P* < 0.05) and relative (*r* = −0.34, *P* < 0.05) changes in the CK activity between PRE and MID. Body weight (*r* = −0.53, *P* < 0.01) and fat percentage (*r* = −0.60, *P* < 0.01) were negatively correlated with the change in the leptin concentration between the PRE and MID conditions. (Table 3).

**Table 3:** Associations between physical performance and body composition and absolute and relative changes in serum biomarkers (*P < 0.05; **P < 0.01)

	IGF‐1 Δ PRE‐MID	IGF‐1 (Δ%) PRE‐MID	Leptin Δ PRE‐MID	Leptin (Δ%) PRE‐MID	CK ΔPRE‐MID	CK (Δ %) PRE‐MID
BW (kg)	−0.056	0.000	−0.531**	−0.265	0.303	0.249
FAT%	−0.024	0.019	−0.604**	−0.194	0.129	0.160
SMM (kg)	−0.047	−0.006	−0.274	−0.217	0.301	0.212
*V*O_2max_ (mL/kg/min)	0.048	0.069	0.078	0.036	0.009	−0.034
SU (reps)	0.142	0.156	0.220	0.139	−0.101	−0.037
PU (reps)	0.355*	0.367*	0.144	0.138	−0.315*	−0.341*
SLJ (m)	−0.054	−0.079	0.154	0.003	0.156	0.123

BW, body mass; FAT%, fat percentage; SMM, skeletal muscle mass; VO_2_max, maximal oxygen uptake; SU, sit‐ups; PU, push‐ups; SLJ, standing long jump; PRE, Premeasurement point; MID, Midmeasurement point.

### Associations between immunological and hormonal concentrations

**Figure 1:**
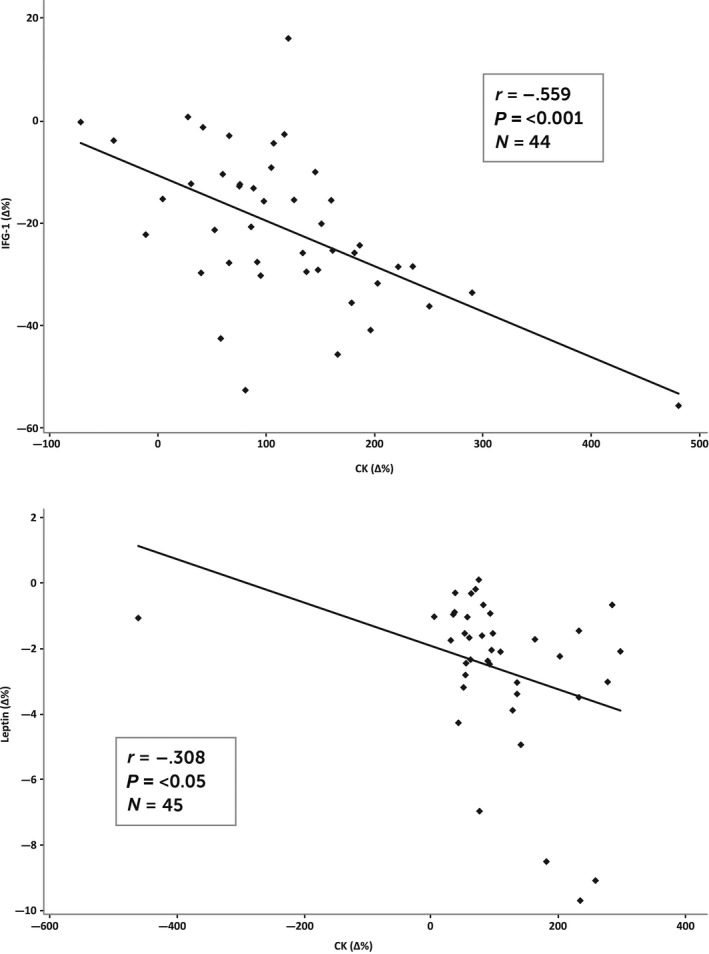
Correlations between the changes in leptin and IGF‐1 compared to the changes in CK between the PRE and MID measurements.

The change in the leptin concentration was negatively correlated (*r* = −0.31, *P* < 0.05) with the change in CK between PRE and MID. In addition, the change in IGF‐1 was negatively correlated (*r* = −0.56, *P* < 0.001) with the change in CK between the PRE and MID measurements (Fig. 1).

## Discussion

The present results show that the prolonged MFT caused changes in conscripts’ hormonal and immunological values and body composition. There was a decrease in the IGF‐1 and leptin concentrations. Body weight, fat mass and skeletal muscle mass decreased at the same time. The main finding of the study was that upper body dynamic strength and endurance were associated with the changes in most serum biomarkers during the prolonged MFT. On the contrary, the serum TNF‐*α* and IL‐6 concentrations did not change during MFT. Furthermore, there was no association between aerobic endurance and the changes in hormonal and immunological concentrations during MFT. The results show that upper body muscle endurance had a significant impact on conscripts’ strain in the battlefield.

During MFT, the conscripts served their last weeks of the compulsory military service. Their maximal oxygen uptake was 49.2 ± 4.8 mL/min/kg, which is comparable with US soldiers (Sharp et al. 2008), and higher than an average value in the Finnish army after the basic training period (Santtila et al. 2008). During the present study, the subjects took 12165 ± 2381 steps per day, however, there was a high variation in their daily activity. The amount of activity was the highest during the first 14 days of MFT and lowest during the final 8 days. It was higher than the previously reported values in German soldiers (Schulze et al. 2015) and Norwegian Home Guard troops (Aandstad et al. 2016), but lower than during the US Army basic combat training (Knapik et al. 2007). During the prolonged MFT, the conscripts lost 2.3% of their body weight, 7.7% of their fat mass and 2.3% of their muscle mass between the PRE and POST measurements. In the RECO measurements, all the body composition variables were almost the same as in the PRE measurements. This suggests that the loss of body weight was mainly due to dehydration. Similar results have also been reported in previous studies (Nindl et al. 2002; Chester et al. 2013). According to Kyröläinen et al. (2008) and Margolis et al. (2014) the need for energy intake can be even 5000–7000 kcal/day. Loss of body weight and muscle mass have been shown to lead to decline in aerobic and anaerobic performance (Shippee et al. 1994; Montain and Young 2003). Loss of body mass has also been shown to lead to a decline in muscle strength of the lower extremities but not in grip strength (Johnson et al. 1994; Montain and Young 2003). In addition, loss of body mass has been demonstrated to associate with anabolic and catabolic reactions, controlled by signaling in the muscle cell. IGF‐1 activated signals have, however, been shown to block muscle loss (Gordon et al. 2008). It was found that the IGF‐1 concentration decreases during MFT, which can lead to loss of muscle mass. In the present study, the bioelectrical impedance analysis was utilized to measure changes in body composition, although it has been shown to overestimate muscle mass and body fat (Sillanpää et al. 2014). It is important to notice that when measuring with BIA, fluid shifts and hydration status may have led to the differences observed in body composition. In future studies, it is recommended that measurements apparatus such as DEXA (Dual X‐ray Absometry) should be used to measure body composition more accurately.

The serum IGF‐1 concentration decreased by 21.5% during MFT but recovered after 4 days almost back to the PRE‐level. Similar findings have also been found in previous studies (Nindl et al. 2002; Rosendal et al. 2002; Vaara et al. 2015). Larger decreases (62%) have been found during the US Army Ranger course (Nindl et al. 2007). The decrease in the serum IGF‐1 concentration might be due to prolonged physical strain and energy deficit during MFT when energy intake, especially protein intake, is not sufficient enough (Friedl et al. 2000). Respective decreases in IGF‐1 have also been found after a strenuous sport event (Eliakim et al. 2005). At a microlevel, the reason for decrease in IGF‐1 might be caused by the activation of AMP‐kinase, which has been shown to activate after low intensity exercises, like marching accompanied with an energy deficit. AMP‐kinase has opposite interactions to cell function than IGF‐1, and it is possible that it might inhibit the IGF‐1 signal route in the muscle cell. Previous studies have shown that the AMP‐kinase inhibits protein synthesis in the muscle cell and increases the breakdown in muscles (Hardie 2004; Wadley et al. 2006). A decrease in serum IGF‐1 has an adverse effect on soldiers because it shows that the body's ability to repair muscle damage and recover from physical strain is impaired and that can lead to an overreaching state.

The serum TNF‐*α* concentration was the highest in the MID measurement. Previous studies have not shown consistent findings with the effects of physical strain on TNF‐*α* concentrations. It appears that it is affected by the duration and intensity of training and subjects training age (Dufaux and Order 1989; Espersen et al. 1990; Rivier et al. 1994). Nindl et al. (2012) studied the changes in TNF‐*α* during a 4‐month basic training period and found a decrease in TNF‐*α*. Increases in TNF‐*α* concentration has been suggested to be due to the inflammation state in the body, caused by muscle damage (Espersen et al. 1990). Rämson et al. (2008) found that the increase in the TNF‐*α* concentration was due to increased fat metabolism caused by an energy deficit. It is likely that during the first 14 days of MFT, hard physical strain and energy deficit have increased TNF‐*α*. When the physical strain has decreased, also serum TNF‐*α* has decreased. The results of the present study show that the serum TNF‐*α* concentration could be an applicable tool for evaluate warfighters’ physical state and recovery.

The CK activity increased by 88% between the PRE and MID measurements and decreased after that in the POST and RECO measurements. The increase in CK is believed to be account of muscle adaptation to eccentric work (Kyröläinen et al. 2008). When comparing to previous studies (Kyröläinen et al. 2008; Chester et al. 2013; Margolis et al. 2014), the increase in CK in our study was quite small. Previous investigations have been more intensive with longer marches, more high intensive runs but shorter study duration than in the present study. The results of this study provide a better understanding on longer duration and low intensity MFT on the CK activity in soldiers. Although the changes were relatively small, the changes show us the difference in physical load in several stages of MFT. It seems that monitoring the CK activity could be a helpful marker when evaluating warfighters’ physical strain during MFT.

The serum leptin concentration decreased by 66% from the PRE to MID measurements but recovered in the RECO measurements. This supports Gomez‐Merino et al. (2002), who found that leptin concentration decreased by 73% in a 5‐day MFT. Energy deficit has been shown to be an influencing factor for the decreased leptin concentration (Landt et al. 1997; Perusse et al. 1997). It can be interpreted that the decrease in leptin was due to the energy deficit in the present study. It is also notable that the change in the leptin concentration associated negatively with the change in the CK activity, which shows that the change in the leptin concentration may be related to muscle damage.

In the present study, no significant differences in the IL‐6 concentrations were observed during the study period due to large individual variation. Chester et al. (2013) and Nindl et al. (2012) have also reported similar findings previously. Gomez‐Merino et al. (2002) and McClung et al. (2013) have found, however, increases in the IL‐6 concentrations during prolonged MFT.

This investigation found that the number of push‐ups performed in one minute and the relative changes in the serum IGF‐1 concentration and CK activity between the PRE and MID measurements were associated with each other. However, no other physical performance factors associated with the measured serum biomarkers. The results show that upper body strength and endurance is related with the physical strain during prolonged MFT. Previous studies (Harman et al. 2008; Knapik and Reynolds 2011) are in line with the results of the present study. Harman et al. (2008) found that upper body strength correlated with 400 m and 30 m run and an obstacle course. Knapik and Reynolds (2011) studied muscle strain during a foot march with heavy load carriage and found that the upper body muscles were loaded more than the lower body muscles. According to these findings, it is important to have enough muscle endurance capacity in the upper body for succeeding well in the ordered tasks.

## Conclusion

The present study showed that there were negative changes in warfighters’ body composition and hormonal and immunological values during a prolonged MFT. These changes suggest that the physiological stress was high during MFT. It is important, especially, during a prolonged MFT, to have sufficient energy intake to prevent negative physiological effects from developing. A good level of upper body and trunk muscle endurance negatively correlated with warfighters’ physiological effects. Thus, it is important to develop warfighters’ upper body strength and muscular endurance to prevent negative physiological effects.

## Conflict of Interest

None declared.
